# Evidenzbasierte Alkoholprävention – Was empfiehlt die Wirksamkeitsforschung? Ergebnisse der BZgA-Expertise zur Suchtprävention 2020

**DOI:** 10.1007/s00103-021-03342-9

**Published:** 2021-05-31

**Authors:** Anneke Bühler, Johannes Thrul, Elena Gomes de Matos

**Affiliations:** 1 Hochschule für angewandte Wissenschaften Kempten, Bahnhofstr. 61, 87435 Kempten, Deutschland; 2 Johns Hopkins University, Baltimore, USA; 3 IFT Institut für Therapieforschung, München, Deutschland

**Keywords:** Alkohol, Prävention, Wirksamkeit, Review, Jugend, Alcohol, Prevention, Effectiveness, Review, Youth

## Abstract

**Hintergrund:**

Der riskante Alkoholkonsum einer substanziellen Gruppe von Jugendlichen und jungen Erwachsenen weist auf einen alkoholpräventiven Handlungsbedarf hin. Die BZgA-Expertise zur Suchtprävention 2020 liefert das wissenschaftliche Wissen zur Wirksamkeit von suchtpräventivem Handeln mit jungen Menschen.

**Fragestellung:**

Welche Ansätze haben in den jeweiligen Handlungsfeldern der Suchtprävention alkoholpräventive Effekte?

**Methode:**

Eine systematische Literatursuche im Juni 2017 in 7 internationalen Datenbanken resultierte in 28.949 Treffern. Einschlusskriterien waren: Studientyp Review oder Metaanalyse, Erscheinungsdatum 2012–2017, Zielgruppe universell oder selektiv und Alter bis 25 Jahre, Zielverhalten Alkoholkonsum. Ausschlusskriterien waren: Zielgruppe Menschen mit diagnostizierten Störungen, Zielverhalten Risikofaktoren. 34 alkoholbezogene Arbeiten wurden von den 3 Autoren systematisch ausgewertet und mittels AMSTAR (A MeaSurement Tool to Assess systematic Reviews) methodisch bewertet. Im Konsensverfahren wurden Schlussfolgerungen und Empfehlungen formuliert.

**Ergebnisse:**

Basierend auf 53 Schlussfolgerungen zur Wirksamkeit von Alkoholprävention lassen sich je nach Handlungsfeld (Familie, Schule, Hochschule, Medien, Gesundheitssystem, Kommune) und Zielgruppe u. a. empfehlen: Familienprogramme und Elterntrainings, verhaltensbezogene Programme, die bestimmte personale und soziale Kompetenzen fördern, Kurzinterventionen mit Feedback, Mentorenprogramme. Neuere Arbeiten zu verhältnispräventiven alkoholpolitischen Maßnahmen auf kommunaler oder nationaler Ebene konnten nicht identifiziert werden.

**Diskussion:**

Verhaltensbezogene Alkoholprävention ist wirksam. Je nach Handlungsfeld und Zielgruppe empfiehlt sich ein sehr differenziertes Vorgehen. Benötigt wird ein Konsens, mit welcher Art von Evidenz die kausale Wirksamkeit von Verhältnisprävention nachgewiesen werden kann.

## Abstract (en)

**Background:**

A substantial group of adolescents and young adults engage in risky alcohol use and there is a need for alcohol prevention. The 2020 BZgA review of reviews on addiction prevention provides the best available scientific knowledge on effectiveness of prevention measures for young people.

**Research question:**

Which approaches show preventive effects on alcohol use in different prevention settings?

**Methods:**

A systematic literature search in June 2017 in seven international databases resulted in 28,949 hits. Inclusion criteria were a review or meta-analysis study type, a 2012–2017 publication date, a universal or selective target group, age up to 25 years, and a target behavior of alcohol consumption. Exclusion criteria were target group people with substance use disorders and target behavior risk factors. The three authors performed a systematic content analysis of 34 alcohol-related publications and assessed their methodology using AMSTAR (A MeaSurement Tool to Assess systematic Reviews). Conclusions and recommendations were framed by consensus among all authors.

**Results:**

Based on a total number of 53 conclusions on the effectiveness of alcohol prevention approaches depending on setting (family, school, college, media, health care, and community) and target group, it can be recommended – among other things – to implement family programs and parenting training, behavioral programs targeting specific personal and social skills, brief interventions with feedback, and mentoring programs. No recent reviews that investigated the effectiveness of alcohol policies at the community or national level could be identified.

**Conclusions:**

Behavioral alcohol prevention is effective. It is recommended to address specific age and target groups in different settings by using specific interventions. Consensus is needed with regard to what kind of evidence proves effectiveness of environmental prevention measures.

## Einleitung

Alkohol ist die psychoaktive Substanz, mit der junge Menschen als Erstes, am häufigsten und leicht in Kontakt kommen [1, 2]. Sie verspricht sowohl Gemeinschaftsgefühl und Spaß als auch Unterstützung bei der Bewältigung der Anforderungen und Herausforderungen des Jugendalters [3]. Gleichzeitig sind mit Alkohol die schwerwiegendsten Schädigungen und Probleme des Jugend- und Erwachsenenalters verbunden [4]. Trotz der zu beobachtenden Abnahme des Alkoholkonsums über die letzten Jahrzehnte, konsumiert eine substanzielle Gruppe von Jugendlichen und jungen Erwachsenen Alkohol in einer riskanten Art und Weise [1, 5]. Die sozioökologische Sichtweise weist auf die multikausal bedingte Entwicklung von Alkoholkonsum und -missbrauch hin [6]. Einflussreiche Risiko- und Schutzfaktoren sind in allen Mikrosystemen jugendlicher Entwicklung sowie auf Makroebene empirisch identifiziert worden [6, 7].

Einen verantwortungsvollen Konsum in einem bezüglich Alkohol relativ liberal regulierten Land wie Deutschland [4] zu erlernen ist herausfordernd. Seine Entwicklung kann mit zielgerichteten verhaltensbezogenen Präventionsprogrammen und mit der verhältnisbezogenen, gesundheitsförderlichen Gestaltung der Lebensbedingungen der jungen Menschen unterstützt werden [8]. Eine evidenzbasierte Entscheidung, wie suchtpräventiv zu handeln ist, verknüpft das bestmögliche wissenschaftliche Wissen mit der Expertise der Praxis und den Besonderheiten der jeweiligen Zielgruppe und des Kontextes [9]. Die Expertisen zur Suchtprävention der Bundeszentrale für gesundheitliche Aufklärung (BZgA) liefern das aktuelle wissenschaftliche Wissen zur Wirksamkeit von Angeboten in unterschiedlichen Handlungsfeldern der Suchtprävention [10, 11].

### Ziel und Adressatenkreis der BZgA-Expertise zur Suchtprävention 2020

Ziel der Expertise ist es, die Wirksamkeit existierender suchtpräventiver Ansätze anhand von aktuellen, hochwertigen wissenschaftlichen Studien zu beurteilen. Wirksamkeit wird dabei definiert als Verhinderung, Verzögerung oder Reduktion des Konsums von Tabak, Alkohol, Cannabis und anderen illegalen psychoaktiven Substanzen. Ebenso wird die derzeitige Prävention des problematischen Glücksspielverhaltens bewertet. In Augenschein genommen werden verhaltens- und verhältnisbezogene Präventionsformen, darunter universelle und selektive Strategien. Universelle Programme richten sich an Personen, die als Gesamtgruppe ein durchschnittliches Risiko für einen späteren Substanzmissbrauch aufweisen (z. B. Gesamtbevölkerung, Klassenverbände). Selektive Programme richten sich an Personen, die als Gruppe ein überdurchschnittliches Risiko für einen späteren Substanzmissbrauch aufweisen (z. B. Kinder aus suchtkranken Familien, Kinder mit Verhaltensauffälligkeiten). Die aus der Forschung abgeleiteten Aussagen werden jeweils für die unterschiedlichen Handlungsfelder gruppiert: Familie, Schule, Hochschule, Medien, Gesundheitsversorgung, Kommune und gesetzliche Rahmenbedingungen. Innerhalb dieser Handlungsfelder werden die Schlussfolgerungen nach Substanzen getrennt. Ihre Aussagekraft (Evidenzstärke) wird ausgewiesen.

Der Adressatenkreis der Expertise sind Verantwortliche für Suchtprävention (Entscheidungsträger*innen) auf allen handlungspolitischen Ebenen sowie Personen, die mit der Entwicklung und/oder Durchführung präventiver Maßnahmen betraut sind.

Für den folgenden Beitrag wurde der Fokus auf die Wirksamkeit alkoholpräventiver Ansätze begrenzt und die alkoholbezogene Literatur ausgewertet. Die konkrete Fragestellung lautet: Welche Ansätze haben in den jeweiligen Handlungsfeldern der Suchtprävention alkoholpräventive Effekte? Zuerst wird die Methode vorgestellt, die in [11]^1^ ausführlich beschrieben ist. Die darauffolgenden Ergebnisse sind nach Handlungsfeld gruppiert. In der abschließenden Diskussion wird ein Schwerpunkt auf den Vergleich zwischen internationaler und nationaler Evidenz sowie auf die Evidenz für alkoholpolitische Strategien gelegt.

## Methode

### Literaturrecherche und -auswahl

Eine Literatursuche wurde im Juni 2017 in internationalen Datenbanken (Cochrane Library, PsychInfo, DARE, Psyndex, Pubmed, Web of Science und Campbell) durchgeführt und auf Veröffentlichungen beschränkt, die in den Jahren 2012 bis 2017 erschienen waren. Die Suche erfolgte in deutscher und englischer Sprache anhand von Suchbegriffen (hier exemplarisch auf Englisch):

**Substance**: substance, smok*, tobacco, nicotine, alcohol, drug, marijuana, marihuana, cannabis, illicit, ecstasy, amphetamine, psychoactive, inhalant*, solvent, „prescription drugs“, „legal highs“

**Measure**: intervent*, program*, treatment*, campaign, policy, policies, legislation, educat*, promot*, adverti*, counsel*, teach*, school, family, community, workplace

**Target behavior**: use, misuse, abuse, onset, reduc*, prevent*, increas*, decreas*, chang*, cessation, abstain*, stop, intoxicat*, uptake, addict*, initiation

**Evaluation**: evaluat*, success*, effective*, efficacy*, measur*, examin*, compar*, trial*, rct

Type of study: meta-analysis, review

**Figure 1:**
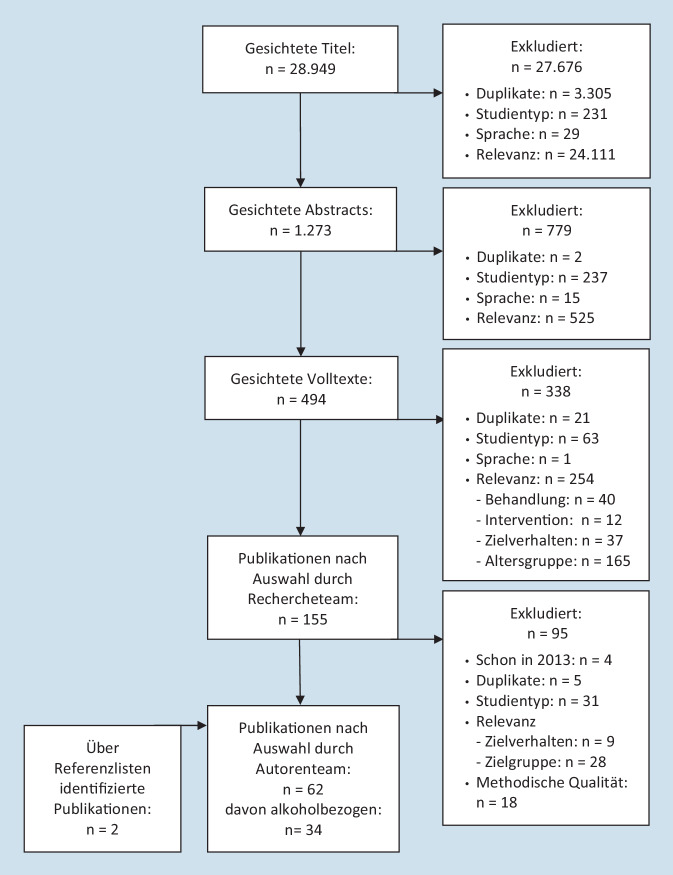

Aus fast 29.000 Treffern der Literaturrecherche und einschlägigen Publikationen wurden systematisch 62 Übersichtsarbeiten ausgewählt (Abb. 1). Arbeiten wurden dann eingeschlossen, wenn folgende Kriterien erfüllt waren: Ziel Prävention, Studientyp Review oder Metaanalyse, Zielgruppe junge Menschen im Alter von bis zu 25 Jahren (universell, selektiv oder indiziert) und Zielverhalten Konsum. Ausgeschlossen wurden Arbeiten, die sich mit der Behandlung von Personen mit bereits diagnostizierten Störungen beschäftigten oder nur Effekte auf kognitive oder soziale Risikofaktoren des Konsums berichteten. Für den vorliegenden Beitrag stellen die 34 Arbeiten mit Alkoholbezug die Basis dar. Die ausgeschlossenen Arbeiten sind im Anhang von [11] inklusive Ausschlussgrund gelistet, die eingeschlossenen Arbeiten mit Bezug zu Alkohol sind in Tab. 1 skizziert.

**Table 1:** 

Handlungsfeld	Arbeit^a^	Anzahl Studien	Art Studien^b^	Zielgruppe: Alter und Ausrichtung^c^	Anzahl erfüllter AMSTAR-Kriterien^d^	Effektstärke Alkoholkonsum^e^
Familie	[13] NR	66	RCT	9–17; U&S	3/5	–
	[14] NR	39	RCT, CT	9–18; U&S	3/5	–
	[15] M	116	RCT, CT, +	11–21; U&S	1/7	d = 0,31 (SK)
Schule	[16] M	228	RCT, CT	1.–12. Klasse; U, S	2/7	d = −0,10. bis d = −0,14
	[17] M	13	RCT, CT	6–12,5; U	4/7	RR = 0,74
	[18] M	19	RCT	5–18; U	7/7	n. s.
	[19] M	28	RCT, CT	M = 15,6; U&S	4/7	g = 0,34
	[20] M	8	RCT	4–18; S	6/7	SMD = bis 0,91
	[21] M	28	RCT	M = 13,2; U	5/7	g = 0,22
	[22] M	25	RCT	12–18; U	5/7	OR = 0,80
	[23] NR	10	RCT, CT	13–17; U&S	3/5	–
Hochschule	[24] M	30	RCT, CT, +	18,6–20,4; S	3/7	d = 0,13 bis d = 0,20
	[25] M	15	RCT, CT, IST	M = 20; S	2/7	d = −0,09 (–)
	[26] M	17	RCT	Unklar; U&S	1/7	OR = 0,79 und 0,96 (n. s.)
	[27] M	73	RCT, CT	≤ 25; S	1/7	g = 0,18
	[28] M	8	RCT	19–21; U	4/7	d = 0,28 bis d = 0,29
	[29] M	70	RCT	Unklar; U&S	7/7	SMD = −0,06 bis SMD = −0,21
	[30] M	14	RCT, CT	19–21; U&S	1/7	d = 0,24 bis d = 0,27
	[31] M	18	RCT	18–21; U&S	4/7	MD = −1,5
	[32] M	41	RCT	M = 19; U	3/7	d = −0,11 bis d = 0,14
	[33] M	48	RCT, CT	19–20; U&S	2/7	d = 0,13 bis d = 0,29
Medien	[36] M	13	RCT	14–24; U&S	2/7	d = 0,18 bis d = 0,19
	[37] NR	12	RCT	11–18; U	1/5	–
	[38] NR	15	RCT	18–26; U&S	3/5	–
	[39] NR	8	RCT, CT +	11–18; U&S	1/5	–
Gesundheitsversorgung	[41] M	8	RCT	13–25; S	6/7	Bis zu SMD = 0,17
	[42] M	32	RCT	18–25; S	4/7	d = 0,18
	[43] NR	7	RCT	12–25; S	4/5	–
	[44] NR	5	RCT	16–35; S	3/5	–
Kommune	[45] M	13	RCT, CT	11–18; U	4/7	d = 0,05 (n. s.)
	[46] M	6	RCT, CT	12–19; S	7/7	SMD = 0,16 (SK)
	[47] M	6	RCT	10–16; S	6/7	OR = 0,72
Übergreifend: Kurzintervention	[35] M	313	RCT, CT	11–25; U, S	5/7	g = 0,19 bis g = 0,27
	[34] M	77	RCT	15–24; U, S	7/7	SMD = −0,11 bis SMD = −0,14

^a^*NR* narrativer Review, *M* Metaanalyse

^b^*RCT* randomisiert kontrollierte Studie, *CT* kontrollierte Studie, *ITS* Zeitreihenstudie

^c^Alter in Jahren, *U* universell,* S* selektiv, *U&S* keine Trennung zwischen universell und selektiv

^d^AMSTAR(A MeaSurement Tool to Assess systematic Reviews)-Hauptkriterien: Protokoll, Literaturrecherche, Erklärung für Ausschluss von Einzelstudien, Risikobewertung der Einzelstudien, Interpretation vor Risikobewertung, bei Metanalyse auch Methode der Metaanalyse, Berücksichtigung des Veröffentlichungsbias

^e^Gesamteffektstärken auf Alkoholkonsum in Metanalyse: *d* Cohens d, *g* Hedges g, *SMD* standardisierte Mittelwertdifferenz, *RR* relatives Risiko, *OR* Odds Ratio, (*SK*) Substanzkonsum inkl. Alkohol, *n.* *s.* nicht signifikant, *(–)* negativer Effekt, *+* und weniger aussagekräftige Studiendesigns

### Auswertung

Die Autoren werteten die Arbeiten jeweils eines oder mehrerer Handlungsfelder systematisch aus. Für die inhaltliche Auswertung wurde dabei ein in den vorherigen Expertisen bewährtes Raster verwendet. Der Ergebnisparameter der Expertise ist das Konsumverhalten, nicht kognitive oder soziale Einflussfaktoren des Konsums. Somit wurden nur Arbeiten eingeschlossen, die Aussagen zum lebenszeitbezogenen oder aktuellen Alkoholkonsum erlauben. Diese können dann die Frequenz, die Menge und/oder ein risikoärmeres bzw. riskantes Konsummuster betreffen. Von „präventiven Effekten“ wurde dann gesprochen, wenn in der Interventionsgruppe im Vergleich zu einer Kontrollgruppe weniger Konsum oder ein späterer Konsumeinstieg beobachtet wurde. Um die methodische Qualität der Arbeiten zu beurteilen, kam AMSTAR (A MeaSurement Tool to Assess systematic Reviews) zum Einsatz, ein Instrument, das extra für Übersichtsarbeiten entwickelt wurde [12]. Als besonders relevant werden 7 Kriterien bezeichnet: Studienprotokoll vorhanden, Literaturrecherche systematisch, Ausschluss von Einzelstudien begründet, Risiko der Einzelstudien bewertet, Ergebnisse vor dem Hintergrund der Risikobewertung interpretiert; im Fall einer Metanalyse sollen die Methoden statistisch adäquat sein und die Analyse getrennt für randomisiert kontrollierte Studien (RCT) und kontrollierte Studien (CT) erfolgen. Der Veröffentlichungsbias in Richtung positiver Ergebnisse sollte bei der Interpretation berücksichtigt worden sein [12]. Bei Unsicherheit wurde im Autorenteam diskutiert und im Konsens über den Einschluss einer Arbeit entschieden.

#### Ableitung von Schlussfolgerungen und Empfehlungen

Aus den Ergebnissen der Übersichtsarbeiten wurden Schlussfolgerungen formuliert. Ergebnisse aus verschiedenen Arbeiten zu gleichen Sachverhalten wurden in eine Aussage integriert. Bei Unsicherheit wurde im Autorenteam diskutiert und über die Formulierung einer Schlussfolgerung entschieden. Jede Schlussfolgerung erhielt eine Evidenzstärkebewertung und den Verweis auf die zugrunde liegenden Übersichtsarbeiten. Die Qualität der jeweiligen Evidenzbasis floss in die Formulierung der Schlussfolgerung mit ein. So wurde z. B. formuliert: „hat präventive Effekte“, wenn es sich um eine Aussage handelt, die auf dem positiven Ergebnis einer Metaanalyse mit mindestens 5 hochwertigen Studien (RCT, CT) beruht. Dagegen wird davon gesprochen, dass ein Ansatz „präventive Effekte haben kann“, wenn von Ergebnissen einer Metaanalyse mit weniger als 5 Studien oder eines narrativen Reviews abgeleitet wird. Um die zahlreichen Schlussfolgerungen nochmals zu verdichten, wurden im Rahmen der inhaltlichen Diskussion Empfehlungen herausgearbeitet. Die Methode ist in [11] ausführlich beschrieben. Das Studienprotokoll wurde bei PROSPERO (International Prospective Register of Systematic Reviews) registriert.

In dem Bestreben, das jeweils aktuelle Wirksamkeitswissen zu berichten, wurde im Fall keiner neu identifizierbaren Veröffentlichung auf die Arbeiten und damit Ergebnisse der vorangegangenen Expertise [10] zurückgegriffen, in der die gleiche Methode verwendet worden war.

## Ergebnisse

Bezüglich der Wirksamkeit von alkoholpräventiven Maßnahmen konnten insgesamt 34 und für die jeweiligen Handlungsfelder der Prävention die folgende Anzahl an Übersichtsarbeiten identifiziert werden: Familie 3, Schule 8, Hochschule 10, Medien 4, Gesundheitsversorgung 4, Kommune 3 und gesetzliche Rahmenbedingungen 0 (Tab. 1). 2 weitere Arbeiten stehen für die Beurteilung der Kurzintervention handlungsfeldübergreifend zur Verfügung. 8 Arbeiten werten Einzelstudien zu universeller Prävention, 10 zu selektiver Prävention, 16 zu universeller und selektiver Prävention aus. Die methodische Qualität der Arbeiten liegt im mittleren Bereich. So beträgt der Median der erfüllten AMSTAR-Hauptkriterien für die 24 Metaanalysen bei Med = 4 (von 7) und für die narrativen Reviews bei Med = 3 (von 5). Allgemein sind die Gesamteffektstärken der Metaanalysen als klein einzuordnen. Insgesamt wurden 53 Schlussfolgerungen in Hinblick auf alkoholpräventive Ansätze gezogen.

Im Folgenden werden die Ergebnisse der Auswertung pro Handlungsfeld zusammenfassend berichtet: teilweise in Form von Schlussfolgerungen, aus Platzgründen teilweise in Form von Empfehlungen. Davor wird jeweils die Literaturbasis genannt und die alkoholpräventive Arbeit im jeweiligen Handlungsfeld konkretisiert.

### Familie

2 systematische Reviews [13, 14] und eine Metaanalyse [15] analysieren die Wirksamkeit familienorientierter alkoholpräventiver Angebote. Diese zielen meist darauf ab, Eltern in ihrem Erziehungsverhalten und ihrer elterlichen Selbstwirksamkeit zu stärken und die familiäre – auch alkoholbezogene – Kommunikation zu verbessern. Konkret wird z. B. trainiert, im Rahmen eines autoritativen Erziehungsstils Regeln einzuführen und durchzusetzen. In Familienprogrammen wird sowohl mit Eltern als auch mit Jugendlichen bezüglich ihrer Lebenskompetenz und der gesamten Familie bezüglich Kommunikation und Freizeitgestaltung gearbeitet. Die Auswertung der 3 Übersichtsarbeiten führt zur Schlussfolgerung: „Familienorientierte Prävention kann präventive Effekte auf den Alkoholkonsum Jugendlicher haben.“ Sowohl kurz- als auch langfristig berichtet eine signifikant größere Anzahl der 34 Einzelstudien präventive Effekte von familienorientierten Angeboten als keine präventiven Effekte [13]. Die analysierten Elterntrainings zeigen in den Studien wiederholt signifikante Effekte auf den Alkoholkonsum, in der Regel jedoch nur für einzelne Untergruppen, Konsumparameter oder bestimmte Studienbedingungen [14]. In der Metaanalyse, die allerdings keine systematische Literaturrecherche berichtet, erreichen alle 116 eingeschlossenen Studien eine Gesamteffektstärke von d = 0,31 [15].

### Schule

7 Metaanalysen [16–22] und ein systematischer Review [23] untersuchen Effekte von schulbasierter Alkoholprävention. Prävention an Schulen kann u. a. Wissensvermittlung, das Training sozialer Kompetenzen, die Sensibilisierung für soziale Einflussnahme beinhalten oder die Form gesunder Alternativangebote annehmen. Aus der Vielzahl an Sekundärstudien sticht die Metaanalyse von Onrust et al. [16] hervor, die nicht nur die größte Studienanzahl in ihre Arbeit einschließen, sondern neben der Bestimmung einer Gesamteffektstärke auch besonders effektive Programminhalte mittels Metaregression identifizieren. Sie machen spezifische Aussagen zur Wirksamkeit je nach universeller und selektiver Prävention, nach Altersgruppe, nach Inhalten und nach Substanz. Für die Alkoholprävention werden 154 Programme ausgewertet. Insgesamt ergibt sich eine kleine Gesamteffektstärke bezüglich der universellen Alkoholprävention in den 2 jüngeren Altersgruppen von d = −0,14 und d = −0,10. Bezüglich der selektiven Prävention sind Effekte auf den Alkoholkonsum bei Sechst- und Siebtklässler*innen sowie Zehnt- bis Zwölftklässler*innen (d = −0,10 und d = −0,32) nachweisbar. Aufgrund dieser Arbeit sind sehr differenzierte Aussagen möglich, die sich mit anderen Metaanalysen verknüpfen lassen, die auf sogenannte Resilienz fördernde Angebote [18], auf deutsche Lebenskompetenzprogramme [17] oder den Einsatz von Peers als Vermittler [18] fokussieren. So werden aufgrund der wichtigsten Schlussfolgerungen empfohlen universelle alkoholpräventive Programme mit Schüler*innen aus Grundschule und Unterstufe, die personale und soziale Lebenskompetenzen fördern [16, 17], darunter v. a. Selbstkontrolle und Problemlösekompetenz [16], ergänzt um das Angebot gesunder Alternativen, Elternarbeit und Peeredukation [16, 22].

Allgemein sind in der universellen schulischen Prävention die Aufklärung über Risiken und die Selbstwertförderung allein keine hinreichend wirksamen Inhalte [16]. Die Förderung von Selbstkontrollstrategien stellt sich als sehr bedeutend dar [16]. Zudem stellt man eher Effekte darauf fest, wie häufig und wie viel Alkohol getrunken wird, und nicht, ob überhaupt getrunken wird oder nicht [21].

Auffällig ist das Ausbleiben von Effekten bei Bemühungen in der Altersgruppe der Acht- und Neuntklässler*innen, was sich auch in der internationalen Forschung widerspiegelt [16]. Weder wird die Gesamteffektstärke für diese Altersgruppe signifikant noch lassen sich in der Metaregression oben beschriebene theoretische Ansätze und Inhalte ausmachen, die mit einer überdurchschnittlichen Effektstärke einhergehen [16]. Dagegen finden sich für ältere Schüler*innen (Stufe 10 und höher) wieder wirksame selektive Präventionsansätze, in denen es um Selbstregulation und soziale Normen geht [16] oder die motivationsfördernde kurze Einzelinterventionen umsetzen [16, 19, 20].

Für die Gruppen mit erhöhtem Risiko für eine Suchtentwicklung erweist es sich auch als sinnvoll, bereits in der Grundschule an der Verhaltensregulation zu arbeiten oder Eltern in ihrem regulativen Erziehungsverhalten zu stärken [16]. Ein narrativer Review stellt fest, dass Programme in Schulen, die nicht den Regelschulen zugeordnet werden, auch wirksam sein können [23].

### Hochschule

Die Wirksamkeitsforschung im Handlungsfeld Hochschule hat ihren Schwerpunkt auf der Alkoholprävention und dem Ansatz der Kurzintervention, v. a. in Form von personalisiertem und normativem Feedback sowie auch motivierender Gesprächsführung. Den Schlussfolgerungen liegen Ergebnisse von 10 Metaanalysen zugrunde [24–33]. Zusammengefasst liegt Evidenz vor für die Wirksamkeit von

- universellen alkoholpräventiven Kurzinterventionen mit personalisiertem und normativem Feedback, sowohl persönlich als auch computerbasiert umgesetzt [28, 29, 32],
- universellen und selektiven Angeboten zur Überprüfung von Wirkerwartungen an den Alkoholkonsum [30],
- Screeningverfahren, um Studierende mit erhöhtem Risikoprofil zu erkennen, plus darauffolgende Kurzintervention [31].

Im direkten Vergleich zwischen persönlichem und computerbasiertem Vorgehen, die beide präventive Effekte aufweisen, schneiden die persönlichen Kurzinterventionen etwas besser ab [29, 33]. Weitere sehr spezifische Schlussfolgerungen [24–27, 32] werden hier aus Platzgründen nicht erläutert.

Angesichts der vielen Arbeiten, die für dieses Setting und für den Ansatz der Kurzintervention zur Verfügung stehen, überrascht es nicht, wenn Einschätzungen zu deren Nutzen unterschiedlich sind [34, 35]. Der Konsens scheint darin zu bestehen, dass diese Kurzinterventionen signifikante, wenn auch geringe Effekte auf den Alkoholkonsum und alkoholbezogene Probleme haben.

### Medien

Für das Handlungsfeld Medien untersuchen eine Metaanalyse [36] und 3 systematische Reviews [37–39] die alkoholpräventive Wirksamkeit von internet- und computergestützten universellen Präventionsprogrammen sowie selektiven Kurzinterventionen. Neuere Überblicksarbeiten zu massenmedialen Kampagnen wurden nicht gefunden. Reviews zu neuartiger digitaler Prävention sind rar, konkret konnte nur eine Arbeit über pädagogisch wertvolle Computerspiele (Serious Games) in der Suchtprävention ausgewertet werden. Die universellen Programme, die meist im Schulsetting angeboten werden, sind teilweise computerbasierte Curricula, die zielgruppengerechte Edutainmentelemente, wie z. B. Comicstrips, beinhalten oder virtuelle Berateravatare [37]. Von 9 Programmen weisen 6 hier kleine positive Effekte auf den Substanzkonsum, darunter auch Alkohol, auf [37]. Computerbasierte, selektive Kurzinterventionen haben auch außerhalb des Settings Hochschule präventive Effekte [36]. Präventive Effekte können dabei auch schon in 15 min Kurzintervention erreicht werden [38]. In einem Serious Game navigiert man z. B. virtuell durch ein Stadtviertel, das realistische Hindernisse und Ablenkungen bereithält, welche durch Avatare verursacht werden. Die Avatare werden so generiert, dass sie der spielenden Person ähnlich sind. Es müssen Aufgaben gelöst werden anhand einer zuvor vermittelten Problemlösestrategie [40]. Der narrative Review stellt für 1 der 8 bewerteten Spiele kurzfristige Effekte auf das Konsumverhalten fest, sodass bisherige Befunde zwar Wirkung in Hinblick auf Wissen und kritische Einstellung nahelegen, mehr als das Potenzial zur Verhaltensbeeinflussung aber nicht konstatiert werden kann [39].

### Gesundheitsversorgung

Die Forschung zu Alkoholprävention im Handlungsfeld Gesundheitsversorgung hat zugenommen und 2 Metaanalysen [41, 42] sowie 2 systematische Reviews [43, 44] werten die Einzelstudien mit unterschiedlichem Fokus aus. Analysiert wird die Wirksamkeit von Kurzinterventionen als selektive Strategie, d. h., die untersuchten Stichproben bestehen meist aus Jugendlichen oder jungen Erwachsenen, die aufgrund eines alkoholassoziierten Vorfalls in der Notaufnahme einer Klinik vorstellig werden. Auf Basis der Forschungsergebnisse wird geschlussfolgert, dass selektive alkoholbezogene Kurzinterventionen in Notaufnahmen Effekte auf den Alkoholkonsum von jungen Erwachsenen haben [35, 42]. Dies kann auch für Jugendliche so sein [35]. Auf das Konzept der motivierenden Gesprächsführung, das klientenzentriert und direktiv arbeitet und mittels bestimmter Techniken die Ambivalenz gegenüber dem Konsum verstärken und damit zur Verhaltensänderung motivieren will, wird hier ein besonderer Fokus gelegt [41, 43, 44].

### Kommune

Im Hinblick auf die kommunale Suchtprävention, die koordiniert und partizipativ lokale Projekte mit Maßnahmen in mehreren Handlungsfeldern auf- und umsetzt, konnten für die Aktualisierung der Expertise keine neuen Übersichtsarbeiten gefunden werden. Gleiches gilt für kommunalpolitische Regelungen sowie für Aktivitäten im Partysetting. Somit können hier keine neueren Schlussfolgerungen als in der vorangegangenen Expertise gezogen werden, welche in diesen explizit alkoholpräventiven Maßnahmen ein präventives Potenzial erkannten [10]. In einer neuen Metaanalyse [45] wurde geprüft, inwiefern unspezifische, allgemein entwicklungsorientierte Projekte, die dem gemeinwesenorientierten Konzept der positiven Jugendentwicklung zugerechnet werden können, alkoholspezifische präventive Effekte aufweisen. Es waren keine präventiven Effekte auf den Alkoholkonsum nachweisbar, d. h., die Metaanalyse mit 6 Studien ergab keine signifikante Gesamteffektstärke [45]. Neuere Arbeiten gibt es zur Wirksamkeit von Mentorenprogrammen, die im kommunalen Raum angesiedelt sind. Diese sehen den Aufbau einer langfristigeren persönlichen Beziehung zwischen einem Kind oder Jugendlichen und einer älteren, außerfamiliären Bezugsperson vor, die keine Fachkraft ist. Präventive Wirkung wird dadurch erwartet, dass die Kinder sich mit den Erwachsenen identifizieren, von deren Vorbild lernen, informiert und ggf. fürsprechend in ihren Anliegen gegenüber Dritten unterstützt werden. Durch die erfahrene emotionale Unterstützung und Freundschaft sollen die Selbstwirksamkeit, das Selbstvertrauen und das Gefühl, für andere wichtig zu sein, gefördert werden [46]. Die entsprechenden Metaanalysen ermittelten aus 2 Studien einen signifikanten Gesamteffekt auf den Alkoholkonsum [47] bzw. aus 6 Studien einen signifikanten Effekt auf den Substanzkonsum inklusive Alkoholkonsum [46]. Insofern kann abgeleitet werden, dass selektive Mentorenprogramme präventive Effekte auf den Alkoholkonsum haben können.

### Gesetzliche Rahmenbedingungen

Schließlich gibt es auch bezüglich der Wirksamkeit von gesetzlichen Rahmenbedingungen keine neuen Erkenntnisse, die durch die Methode der Expertise – Übersichtsarbeiten auszuwerten – generiert werden konnten. Bei der Literaturrecherche konnten 4 alkoholbezogene Arbeiten identifiziert werden, die potenziell für das Handlungsfeld der Verhältnisprävention relevant waren. Diese entsprachen aber nicht den Einschlusskriterien, weil sie u. a. keine spezifischen Aussagen zum Trinkverhalten von jungen Menschen machten [48–50] oder methodisch intransparent waren [51]. So werden im Rückgriff auf die vorangegangene Expertise weiterhin Strategien empfohlen, die zu einer allgemeinen Preiserhöhung von Alkoholprodukten führen, eine verstärkte Regulierung, Kontrolle und Sanktion der Abgabe von Alkohol an Minderjährige nach sich ziehen und die Alkoholwerbung einschränken [10].

## Diskussion

Ziel dieses Beitrags war es, die Wirksamkeit beforschter alkoholpräventiver Ansätze mit und für Kinder, Jugendliche und junge Erwachsene anhand von hochwertiger Evidenz zu beurteilen. Dazu wurde auf die Erkenntnisse der BZgA-Expertise Suchtprävention 2020 zurückgegriffen. Diese hat 34 alkoholbezogene internationale Reviews und Metaanalysen systematisch recherchiert und ausgewertet sowie daraus Schlussfolgerungen und Empfehlungen abgeleitet.

Für fast alle Lebenswelten Jugendlicher und damit Handlungsfelder der Prävention lassen sich aufgrund der zurate gezogenen Literatur Ansätze bestimmen, die eine Wirksamkeit erwarten lassen (s. Infobox 1). Die Orientierung an Erkenntnissen zur Wirksamkeit ist ein bedeutender Teil des evidenzbasierten Handelns [9]. Die Aussagekraft der Expertise hat aufgrund der gewählten Methode allerdings auch ihre Grenzen. Forschungsmethodische Limitationen von Reviews und Metaanalysen sind bekannt [12]. AMSTAR ist ein Instrument, um die Güte von Übersichtsarbeiten einzuschätzen [12]. Für die gesamte Literaturbasis der Expertise wurde eine eher mittlere Güte konstatiert. Während die systematische Literaturrecherche und die Risikobewertung der Einzelstudien als Kernkriterium fast in allen Arbeiten umgesetzt worden waren, gab es hinsichtlich der Begründung für den Ausschluss von Einzelstudien noch Verbesserungsbedarf [11].

Praxisorientiert bedeutet die Auswertung von Übersichtsarbeiten unter anderem, nur Aussagen über beforschte Präventionsansätze zu generieren und ein durchschnittliches Urteil über Ansätze und nicht einzelne Angebote oder Maßnahmen fällen zu können. Informationen zur Wirksamkeit von einzelnen Angeboten sind mittlerweile in digitalen Datenbanken erhältlich. Die Datenbanken umfassen in der Praxis bewährte Präventionsangebote (z. B. www.dieinitiative.de) oder sehen einen Begutachtungsprozess vor, der die Effektivität der Programme bewertet in: „Effektivität theoretisch gut begründet“, „Effektivität wahrscheinlich“ und „Effektivität nachgewiesen“ (www.gruene-liste-praevention.de).

Bei der Interpretation der generierten Ergebnisse sollte bedacht werden, dass „wirksam“ hier heißt, dass im Rahmen eines bestimmten Studiendesigns (randomisierte oder kontrollierte Studie) Effekte auf einen begrenzten Ergebnisparameter (Konsumverhalten) nachgewiesen werden konnten. Dies mag als Endparameter oder einziger Indikator kritisch gesehen werden [52], ermöglicht allerdings eine Vergleichbarkeit zwischen den beforschten Ansätzen.

### Vergleich internationale und nationale Evidenz

Eine zusammenfassende, systematische Übersichtsarbeit zur Wirksamkeit von in Deutschland angebotener Suchtprävention steht noch aus. Insofern war es nicht möglich, in der Expertise für Deutschland spezifische Schlussfolgerungen zu ziehen. Einzelne Studienergebnisse lassen sich aber mit der aufbereiteten internationalen Evidenz vergleichen.

Für das Handlungsfeld Familie wird international eine potenzielle Wirksamkeit für alkoholpräventive Familienprogramme festgestellt. In Deutschland wurde kürzlich eine hochwertige Evaluation eines Familienprogramms durchgeführt, deren Ergebnisse sich nicht mit den Schlussfolgerungen der Expertise decken. Mit Familien aus sozial benachteiligten Stadtteilen konnte eine tabakpräventive Wirkung des Programms, nicht aber Effekte auf den Alkohol- und illegalen Substanzkonsum beobachtet werden [53]. Eine nachfolgende Analyse ergab, dass besonders belastete Familien am meisten profitierten [54]. Übereinstimmung findet sich dagegen im Handlungsfeld Schule und Hochschule. Dies zum einen in Hinblick auf die Effektivität von schulbasierten Lebenskompetenzprogrammen [17]. Zum anderen finden sich international Hinweise auf die Wirksamkeit des Ansatzes, Wirkerwartungen zu überprüfen, bei Studierenden. In Deutschland wurden für Schüler*innen, die im Rahmen eines Programms, das die Wirkerwartungen an Alkohol u. a. anhand eines Trinkexperiments und anhand von Übungen zur Perspektivenübernahme hinterfragte, alkoholpräventive Effekte nachgewiesen [55]. Das computerbasierte normative Feedback schließlich hatte, wie im internationalen Kontext beschrieben, ebenso bei deutschen Studierenden alkoholpräventive Effekte [56]. Im Handlungsfeld Gesundheitsversorgung trägt die deutsche Erfahrung mit motivierender Kurzintervention bei Jugendlichen im Kliniksetting zur international uneinheitlichen Einschätzung bei. Arnaud et al. [57] stellten nach 3 und 6 Monaten einen Rückgang des Alkoholkonsums in der Interventions- wie auch in der Kontrollgruppe fest, ohne signifikante Unterschiede zwischen den Gruppen. Damit kann das international überwiegend positive Urteil nicht bestätigt werden.

Dieser Ausschnitt betrachtet einige Kernpublikationen und kann nicht als systematischer Vergleich angesehen werden. Nichtsdestotrotz verdeutlicht er zum einen, dass die deutsche Suchtpräventionsforschung einiges an Evidenz generiert hat. Er macht zudem deutlich, dass es noch weiterer Anstrengung in Form von weiterführenden und replizierenden Studien bedarf. Einzelne Studien sind sicher keine ausreichende Beurteilungsgrundlage, um eine grundsätzliche Entscheidung für oder gegen die großflächige Umsetzung von z. B. Familienprogrammen oder Kurzinterventionen im Kliniksetting zu fällen.

### Verhältnisprävention

Ein neuerer Review zur Wirksamkeit der Einführung von verhältnisbezogenen, alkoholpolitischen Maßnahmen war durch das hier verwendete Rechercheprotokoll nicht zu identifizieren. So wird auf Grundlage der vorangegangenen Expertise weiterhin empfohlen, Maßnahmen zu ergreifen, die den Preis von Alkohol erhöhen, den Zugang und die Verfügbarkeit von Alkohol erschweren und die Alkoholwerbung einschränken [10]. Die Empfehlungen gehen einher mit der Evidenz aus einschlägigen und neueren Beobachtungsstudien, in der nicht nur eine, sondern verschiedene Dimensionen von nationaler und lokaler Alkoholpolitik gemessen und ihr Zusammenhang mit jugendlichem Alkoholkonsum untersucht wurde. Bei den Dimensionen handelt es sich um Regulierungen in Bezug auf die Verfügbarkeit (z. B. Altersgrenze für den Kauf von Alkohol; Verkaufszeiten; lokale räumliche Bardichte; lokale Lizenzierung des Verkaufs unter bestimmten Bedingungen; Alkohol auf lokalen öffentlichen Veranstaltungen), den Trinkkontext (Initiativen zur Sensibilisierung der Bevölkerung; Konsumverbot im öffentlichen Raum; Training des lokalen Verkaufspersonals; Haftung der Gastgeber von Partys für Minderjährige), den Preis (z. B. Besteuerung), die Werbung (Werbeeinschränkungen in den Medien; lokales Verbot von Plakatwerbung, Beschränkungen der Werbung an Verkaufsplätzen) und Alkohol im Verkehr (Alkoholkontrollen, Blutkonzentrationsgrenzen; [7, 58]). So wurde bei einem querschnittlichen Vergleich der Alkoholpolitik von 26 Ländern [7] bzw. von 50 kalifornischen Städten [58] festgestellt, dass je umfassender die staatliche bzw. kommunale Alkoholpolitik ausgeprägt war, desto weniger Jugendliche Alkohol tranken und desto seltener dies taten. Zu nennen sind hier insbesondere die eingeschränkte Verfügbarkeit von Alkoholprodukten und das Alkoholmarketing [7]. Dies sind Dimensionen, die sich auch im längsschnittlichen Design als bedeutsam erwiesen haben, auch in Studien mit jungen Menschen in Deutschland [2, 59, 60]. Ein weiterer verhältnisorientierter Faktor spielt eine auffällige Rolle für den jugendlichen Alkoholkonsum: das durchschnittliche Trinkverhalten der Erwachsenenbevölkerung. Wiederholt scheint er in Studien den Zusammenhang zwischen Alkoholpolitik und jugendlichem Konsum zu vermitteln [58, 61]. Da überrascht nicht, dass die getrennte Analyse von jugendspezifischen und populationsbezogenen Alkoholpolitiken ergab, dass beide Strategien mit weniger Alkoholkonsum von Jugendlichen einhergehen [61]. Eine strengere Regulierung des Alkoholkonsums für die gesamte Bevölkerung könnte demnach, zusätzlich zu jugendspezifischen Maßnahmen, alkoholpräventiv für die jungen Altersgruppen wirken.

Diese verhaltene Formulierung zu präventiven Effekten von Alkoholpolitik („könnte“) liegt an den Designs der Studien, mit denen die Wirksamkeit von Alkoholpolitik überprüft wird bzw. werden kann. Während im verhaltensbezogenen Bereich evidenzstarke Untersuchungspläne in Form einer randomisiert kontrollierten, sogar mehrarmigen Studie möglich und damit häufig sind, lassen sich politische Maßnahmen kaum im Experiment oder isoliert überprüfen. Dies schränkt die Aussagekraft hinsichtlich der Kausalität ein. Allerdings gibt es Ansätze, den Ursache-Wirkung-Zusammenhängen mittels eines umfassenden Methodenmixes näher zu kommen (Hill, 1965 zit. nach [62]). Für den Bereich der Alkoholpolitik haben Sargent und Babor [62] eine Reihe von Reviews vielfältiger Forschungszugänge initiiert, um den Zusammenhang zwischen Alkoholmarketing und Konsumverhalten präzise zu belegen. Ihr Urteil lautet, dass die Kausalität damit bewiesen sei, sie fordern aber gleichzeitig die Wissenschaft auf, über diesen Weg der Generierung von Wirksamkeitsevidenz zu diskutieren.

## Fazit

Angesichts der Frage, wie dem riskanten Alkoholkonsum in der Bevölkerung begegnet werden kann, kann die Präventionswissenschaft Praxis und Politik unterstützen, indem sie belastbare Erkenntnisse für eine wirkungsorientierte Suchtprävention mit Kindern, Jugendlichen und jungen Erwachsenen zur Verfügung stellt. Für jedes Handlungsfeld der Alkoholprävention lassen sich aufgrund des internationalen Forschungsstands Ansätze, Inhalte und Methoden empfehlen (s. Infobox 1). Diese Empfehlungen sollten als Grundlage für das evidenzbasierte Handeln herangezogen werden und ihre Umsetzung vor dem Hintergrund des gesammelten Erfahrungswissens der Praxis und der Besonderheiten der Adressatengruppe und des lokalen Kontexts entschieden werden [9]. Dieser Dreiklang der evidenzbasierten Praxis ist notwendig, da weder „das Rad immer wieder neu zu erfinden“ noch ein „kontextblinder“ Einsatz standardisierter Verfahren zielführend erscheint. Für die Wirksamkeitsforschung stellt sich die Aufgabe, einen Konsens herzustellen bezüglich der Methoden, mit denen die Kausalität des Zusammenhangs zwischen alkoholbezogenen Regulierungen und Konsumverhalten festgestellt werden kann [62].

### Infobox 1 Empfehlungen für die wirkungsorientierte Alkoholprävention

Basierend auf den Ergebnissen in Bezug auf die Wirksamkeit von Alkoholprävention werden folgende Empfehlungen für die (Weiter‑)Entwicklung und Stärkung von wirkungsorientierten Angeboten gegeben. Es gilt:

- Familien und insbesondere Eltern fit zu machen speziell für den Austausch zum Thema Alkohol und die Auseinandersetzungen bei Grenzüberschreitungen sowie allgemein für ein einfühlendes und konsistentes Erziehungsverhalten und ein positives Familienleben;
- in der Schule bei Kindern und jungen Jugendlichen die soziale Kompetenz zu fördern; sie in Selbstkontrolle zu stärken und ihre Entscheidungs- und Problemlösekompetenz zu üben; dabei die Eltern mit einzubeziehen und alternative Freizeitangebote zu schaffen;
- in der Schule ältere Jugendliche aufzuklären und in der Elternarbeit eine klare und konsequent kritische Haltung gegenüber dem Konsum im Jugendalter zu unterstützen;
- bei konsumerfahrenen Jugendlichen, Studierenden und anderen jungen Erwachsenen in Kurzinterventionen zu ermöglichen, die Konsummotivation zu hinterfragen, sich über die eigene Konsummenge bewusst zu werden, und deutlich zu machen, dass riskanter Konsum auch in ihrer Altersgruppe nicht die Norm ist;
- kommunale Ansätze zu verfolgen, in denen koordiniert in mehreren Settings präventiv gearbeitet wird, darunter auch persönliche Kurzinterventionen in Kliniken und Notaufnahmen;
- politisch Rahmenbedingungen zu schaffen, die die Verfügbarkeit von Alkohol und Alkoholwerbung einschränken (Besteuerung von Alkohol, Heraufsetzung der Altersgrenze, Werbeverbot).

## Footnotes

1 https://www.bzga.de/infomaterialien/fachpublikationen/fachpublikationen/band-52-expertise-zur-suchtpraevention-2020/.
